# Sex dimorphism in dioecious Palmer amaranth (*Amaranthus palmeri*) in response to water stress

**DOI:** 10.1007/s00425-021-03664-7

**Published:** 2021-06-29

**Authors:** Mohsen B. Mesgaran, Maor Matzrafi, Sara Ohadi

**Affiliations:** 1 Department of Plant Sciences, University of California, Davis, One Shields Avenue, Davis, CA 95616 USA; 2 Present Address: Department of Plant Pathology and Weed Research, Agricultural Research Organization, Newe Ya’ar Research Center, Ramat Yishay, Israel

**Keywords:** Baker’s Law, Colonization, Flowering synchrony, Gender specialization, Secondary sex characters, Weed management

## Abstract

**Main conclusion:**

Phenological isolation can potentially reduce seed output and may be exploited as a novel tool for ecological management of dioecious weeds.

**Abstract:**

Dioecious plants may benefit from a maximized outcrossing and optimal sex-specific resource allocation; however, this breeding system may also be exploited for weed management. Seed production in dioecious species is contingent upon the co-occurrence and co-flowering of the two genders and can be further disturbed by flowering asynchrony. We explored dimorphism in secondary sex characters in *Amaranthus palmeri*, and tested if reproductive synchrony can be affected by water stress. We have used seeds of *A. palmeri* from California, Kansas and Texas, and studied secondary sex characters under natural conditions and in response to water stress. Seeds of *A. palmeri* from California (CA) and Kansas (KS) were cordially provided by Dr. Anil Shrestha (California State University, Fresno, California) and Dr. Dallas E. Peterson (Kansas State University, Manhattan, Kansas), respectively. Seeds of a third population were collected from mature plants (about 30 plants) from a set-aside field in College Station, Texas. *A. palmeri* showed no sexual dimorphism with regard to the timing of emergence, plant height, and relative growth rate. While the initiation of flowering occurred earlier in males than females, females preceded males in timing of anthesis. Water stress delayed anthesis in males to a greater extent than females increasing the anthesis mismatch between the two sexes by seven days. Our data provide the first evidence of environment-controlled flowering asynchrony in *A. palmeri*. From a practical point of view, phenological isolation can potentially reduce seed output and may be exploited as a novel tool for ecological management of dioecious weeds.

## Introduction

Flowering plants have evolved an unrivaled diversity of sexual systems ranging from selfing hermaphrodites to dioecious species which the separation male and female individuals obligate outcrossing. Unlike animals, most flowering plants are hermaphrodite with unisexual reproduction (dioecy) being rare and only found in ~ 6% of species (Renner 2014). Despite being rare, dioecy has evolved independently from hermaphroditic ancestral state many times (Tanurdzic and Bancks 2004) with ~ 50% of all plant families having ≥ 1 dioecious species (Renner 2014). Similar to many other life history functions, adoption of any sexual strategy is often accompanied by genetic and/or demographic trade-offs. Hermaphroditism (particularly selfing) provides some degree of reproductive assurance and is often associated with the weedy characters [Baker’s Law sensu (Stebbins 1957)], which enables even a single individual to invade a new habitat (Darwin 1876; Baker 1955). Dioecy maximizes outcrossing and thereby reduces the likelihood of inbreeding depression (Thomson and Barrett 1981, 1990; Charlesworth 1999). Dioecy also has the benefit of optimizing the allocation of resources between male and female functions by minimizing the competition between these two functions through sexual specialization, i.e. the so-called “division of labor” theory (Maynard Smith 1978; Charnov 1982).

Despite these advantages, a dioecious breeding system is subject to several demographic handicaps perhaps firstly noted by an Italian botanist, Federico Delpino, as quoted by Darwin (1876): “*Delpino remarks that dioecious plants cannot spread so easily as monoecious and hermaphrodite species, for a single individual which happened to reach some new site could not propagate its kind*”. In addition to above barrier, the two sexes need to flower at the same time and the smaller the degree of temporal overlapping between male and female flowering, the lower the chance of successful fertilization and hence seed production.

We believe that novel strategies can be developed for ecological management of weedy and invasive plants by a better understanding of the above “cracks” in the evolution of a dioecious mating system. Reducing the flowering overlap between the two sexes, (*phenological isolation strategy*) may decrease the chances of successful fertilization and hence seed production. Gender specialization theory suggests that the same environmental cue may trigger differential responses in the two sexes (Lloyd and Webb 1977), hence providing the potential means for phenological isolation.

In many dioecious species, male and female individuals have shown to differ in secondary sex characters (i.e. traits other than the sex itself) related to morphology, physiology, life history and phenology [reviewed in (Lloyd and Webb 1977; Geber and Dawson 1999; Juvany and Munné-Bosch 2015)]. Differences in progression of phenological events (e.g. timing of flowering) can result in temporal segregation of males and females, ultimately reducing the chance of successful cross-pollination in a dioecious population. In fact, most dioecious species seems to be protandrous, i.e. males flower earlier than females (Forero-Montaña and Zimmerman 2010; Forrest 2014). As female’s investment in reproduction is greater than males, it has been hypothesized that females postpone flowering (reproduction) for longer period of resource accumulation (Lloyd and Webb 1977; Purrington and Schmitt 1998). This hypothesis has direct implications for manipulative separation of flowering timing between the two sexes. That is, if requirement for resource accumulation delays flowering in females, then environmental stresses can further isolate the females, exacerbating the flowering mismatch between the two sexes.

In this study, we attempted to test the viability of exploiting the demographic handicap associated with a unisexual breeding system (i.e. phenological isolation) for novel management of dioecious weeds. We used palmer amaranth (*Amaranthus palmeri* S. Watson) as our dioecious model system which has recently been found to have heterogametic male (Montgomery et al. 2019). The species has been ranked as the worst weed in US corn fields in a recent survey (van Wychen 2017) and is rapidly expanding it range in North America and other regions in the world. Moreover, *A. palmeri* has evolved resistance to multiple herbicidal modes of action with 69 herbicide-resistant biotypes found worldwide (Heap 2021). Most of these biotypes were found to be resistant to the widely used herbicide, glyphosate (Roundup^®^), requiring the development of non-chemical methods to control this weed in agricultural and non-agricultural habitats. The pervasive invasion of *A. palmeri* might have come as a surprise to Delpino and Darwin, and certainly is an exception to “Baker’s Law”, who considered dioecy as a barrier to successful colonization (Darwin 1876). *A. palmeri* therefore represents a unique model for studying the association between breeding system and colonization. The objectives of this study were twofold: (1) determine if the male and female individuals of *A. palmeri* differ in secondary sex characters related to growth and development, and (2) test if the environmental stress (drought) can reduce reproduction synchrony between male and female plants by differentially affecting the two sexes.

## Materials and methods

Seeds of *A. palmeri* from California (CA) and Kansas (KS) were cordially provided by Dr. Anil Shrestha (California State University, Fresno, California) and Dr. Dallas E. Peterson (Kansas State University, Manhattan, Kansas), respectively. Seeds of a third population were collected from mature plants (about 30 plants) from a set-aside field in College Station, Texas (TX), in the summer of 2017. Seeds were stored at 4 °C before use. Experiments were conducted in the greenhouse facility (Orchard Parks) of University of California, Davis, during the spring and summer of 2018.

### Experiment 1. Examine secondary sex characters under natural conditions

The aim of this experiment was to examine whether *A. palmeri* has undergone divergent evolution in secondary sex traits related to growth and development. We therefore compared male and female plants of *A. palmeri* for potential differences in emergence pattern, timing of flowering and anthesis, plant height, and relative growth rate (RGR). Using three populations (CA, KS, and TX) and large number of plants (~ 250), *Experiment 1* also served as a platform for a robust estimate of baseline to secondary sex characters in *A. palmeri*, further examined in the second experiment (*Experiment 2*, see below). In July 2018, we sow 200 seeds from each population, into 5 × 5 × 5 cm plastic pots (one seed per pot) filled with a soil mix (1:1:1:3 sand:compost:peat:dolomite). We recorded the emergence time for each individual seed and then transplanted the young plants (two–four-leaf stage) into larger plastic pots (2.37 L) filled with a soil mix (1:1 sand/peat) plus a controlled-release fertilizer (15–9–12, 150 g 75 L^−1^; Scotts Osmocote PLUS, Mississauga, ON). Pots were randomly placed in a net house under natural California summer conditions and watered twice a day through an automated drip irrigation. Plant height was measured three times (11 July, 23 July, and 24 September) during the season. We inspected plants daily and an individual was recorded to be at the flowering stage when the tip of its inflorescence was visible either on the terminal or lateral buds. The onset of anthesis for a flowering plant was then determined when the stigma (in pistillate flowers) or dehisced anther (in staminate flower) become visible.

### Experiment 2. Study flowering synchrony in response to water stress

This experiment aimed at evaluating the effect of water stress on flowering synchrony between the male and female plants of *A. palmeri*. Because of space and seed limitations, we only used two populations (CA and KS) in this experiment. The experiment was conducted twice (April–July and July–September in 2018) in a completely randomized design with 50 replicates (plants) per treatment (water stress *vs.* well-watered control). About 10 seeds were sown in each round plastic pot (2.37 L), filled with a soil mix (1:1 sand/peat) plus a controlled-release fertilizer (15–9–12, 150 g 75 L^−1^; Scotts Osmocote PLUS, Mississauga, ON). Pots were placed in a greenhouse set at a temperature of 32/22 °C (day/night) with a day length of 16 h provided through supplementary lighting between 5:00 to 9:00 a.m. and 5:00 to 9:00 p.m. Seedlings were thinned haphazardly several times to achieve one plant per pot by their four-leaf stage. When plants reached six to seven true-leaf stage, 50 random plants from each population were randomly assigned to well-watered or water-deficit treatments. Well-watered plants received water from four emitters inserted into the potting medium to deliver 65 mL of water min^−1^ for two min and twice per day (7:00 a.m. and 2:00 p.m.). Water-deficit treatment was achieved by removing three out of four drip emitters from designated water stress treatment pots, reducing the amount of water by approximately 75%. Additional irrigation (~ 100 mL twice per week) was added to water-deficit plants when severe visual stress symptoms were observed. Plants were sexed by examining flowers for the presence of anther (in males) or stigma (in females). For each plant, we also measured time to flowering (inflorescences) and time to anthesis as described in *Experiment 1*.

### Statistical analysis

For both experiments, the data from each population were analyzed separately as testing the differences between populations was not our aim in this study. We used multiple populations mainly as biological replicates to examine the generality of our conclusions pertinent to sexual dimorphism. In* Experiment 1*, using a *t* test, male and female plants were compared for the secondary sex characters including time to flowering (inflorescence), time to anthesis, plant height, and early-stage RGR. Function ‘t.test()’ of ‘stats’ package (R Core Team 2020) was used for the *t* test analysis. The early-stage RGR was calculated based on the height gain between the first and second height measurements using the following formula (Radosevich et al. 2007):

1 $${\text{RGR}} = \frac{{\log \left( {H_{2} } \right) - \log \left( {H_{1} } \right)}}{{t_{2} - t_{1} }},$$

where *H*_1_ and *H*_2_ are plant heights measured at time *t*_1_ (11 July) and *t*_2_ (23 July), respectively (log stands for natural logarithm). To explore difference in seedling emergence pattern between the sexes, we fitted the following two-parameter logistic equation to cumulative emergence data of *A. palmeri* using the ‘drm()’ function of ‘drc’ package (Ritz et al. 2016):

2 $$E\left( t \right) = \frac{1}{{1 + \left( {b\left( {\log \left( t \right) - \log \left( {t_{{50}} } \right)} \right)} \right)}},$$

where *E*(*t*) is the cumulative emergence over time, *t*, *t*_50_ is time to 50% emergence while *b* denotes the steepness of emergence curve around *t*_50_. To statistically test the differences in emergence pattern between the two sexes, we first fitted a full model which included sex a as grouping factor in the model. We then fitted a reduce model, lacking the sex as a grouping factor, and compared the size of errors between these two models with an *F* test using ‘ANOVA’ function in R (R Core Team 2020).

In *Experiment 2*, linear mixed models were fitted to examine the effects of water treatment on time to inflorescence and time to anthesisin male vs. female plants using the ‘lmer()’ and ‘ANOVA()’ functions of ‘lme4’ (Bates et al., 2015) and ‘car’ (Fox et al. 2019) packages in R, respectively. Experimental run was incorporated into the mixed model as a random effect (intercept) whilst water treatment, sex, and their interaction were fixed effects. Inspection of residuals showed no predictable pattern when plotted against fitted values suggesting that the ANOVA’s assumption of homogeneity of variance has been met. The degree of flowering/anthesis overlap between the two sexes was visually inspected by fitting a kernel density smoother to time data (i.e. time to flowering and time to anthesis) using the ‘geom_density_ridges()’ function of ‘ggplot2’ R package (Wickham et al. 2019).

## Results

### Experiment 1. Baseline dimorphism in secondary sex characters

#### Seedling emergence

**Fig. 1:**
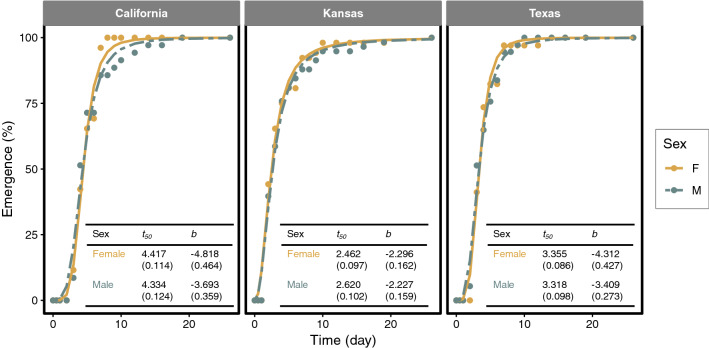
Comparison of cumulative seedling emergence between female (F) and male (M) plants in three populations *A. palmeri* originating. Inset tables show parameter estimates for the logistic model (Eq. 2). Values in the parentheses indicate stand errors. Note that emergence data were normalized relative to the final number of seedlings counted for each sex within the population (see text for definition of model’s parameters)

Total emergence for California, Kansas, and Texas populations were 31%, 55%, and 35% (*n* = 200 for each individual population), respectively. Logistic model (Eq. 2) fitted the emergence data adequately as evidenced in Fig. 1 and small root mean square errors (RMSE ≤ 4%) in all populations. Female and male plants of *A. palmeri* exhibited almost identical emergence pattern and in none of the tested populations, the emergence curves differed between the sexes. Populations, however, differed in emergence rate (rapidity) with Kansas being the fastest emerging population (*t*_50_ = 2.5 ± 0.10 days) while California was the slowest (*t*_50_ = 4.3 ± 0.12 days) irrespective of the gender (see inset in Fig. 1).

### Time to flowering and anthesis

**Fig. 2:**
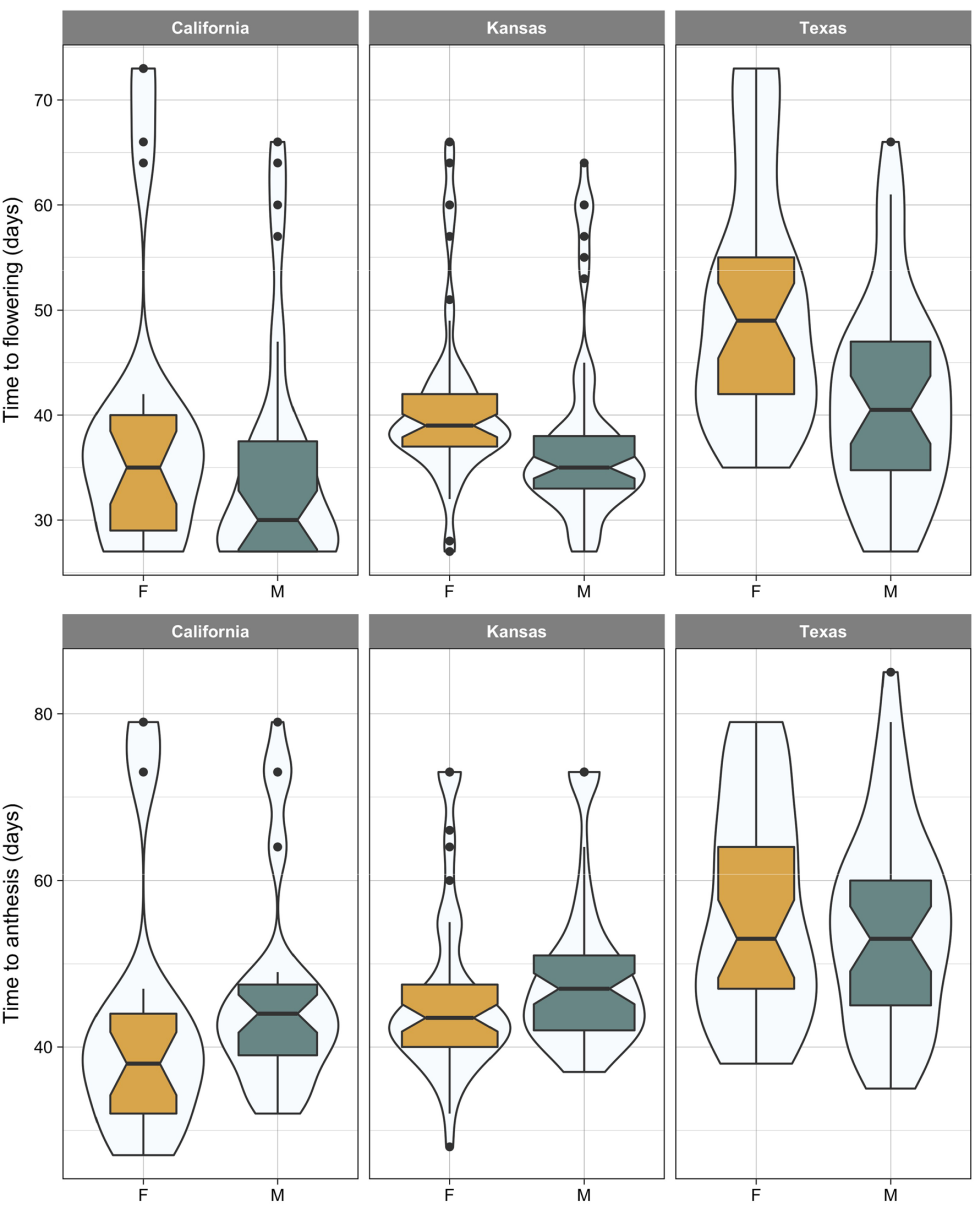
Days to first flowering (inflorescence visible; top row) and anthesis (bottom row) in female (F) and male (M) plants of *A. palmeri* from three populations (California, Kansas, and Texas). The width of violin plot represents the relative frequency of the event of interest (i.e. flowering or anthesis time). Horizontal line within the boxplot shows the median, the box includes the interquartile range, IQR, (i.e. 25th to 75th percentile range), whiskers extend 1.5 times the IQR, and filled circles represent extreme values which fall within > 1.5 × IQR and < 3 × IQR

In all three populations, the initiation of flowering (i.e. tip of inflorescence becoming visible) occurred significantly (Kansas: *p* value = 0.0255, *df* = 108; Texas: *p* value = 0.0011, *df* = 68) earlier in male than female plants of *A. palmeri* (Fig. 2) except for California population (*p* value = 0.2578, *df* = 51). The difference in timing of flowering was particularly profound in Texas population where males, on average, flowered ~ 9 days before females. Variability in flowering time seemed to be similar between males and females as illustrated by the violin plots. The only exception was observed with California population where the flowering bulge occurred close to the earliest flowering times (i.e. at the bottom of the violin) as opposed to females that had their bulge above the median time of flowering. Note that a bulge on a violin plot represents the value (here flowering time) with the highest probability of occurrence.

**Fig. 3:**
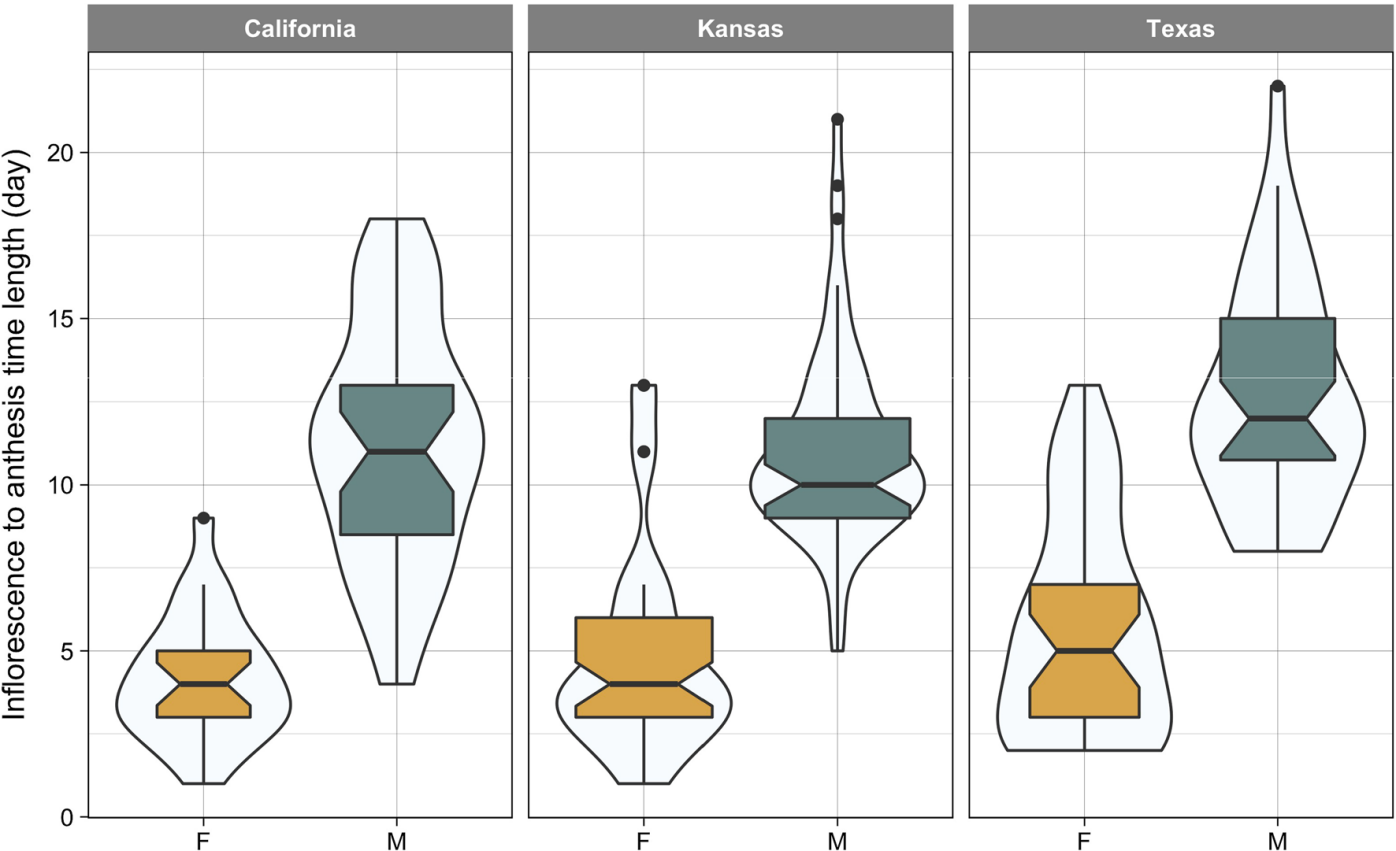
Length of time (day) from flowering (inflorescence visible) to anthesis in female (F) and male (M) plants of *A. palmeri* from three populations (California, Kansas, and Texas). The width of violin plot shows the relative frequency of event (inflorescence to anthesis time). The horizontal line within the boxplot shows the median, the box includes the interquartile range, IQR, (i.e. 25th to 75th percentile range), whiskers extend 1.5 times the IQR, and filled circles represent extreme values which fall within > 1.5 × IQR and < 3 × IQR

Despite marked differential timing of flowering between the two sexes, *t* test showed no significant difference between male and female plants of *A. palmeri* for time to anthesis across the three populations tested in this study (Fig. 3). However, in both, California and Texas populations, anthesis happened 4 days earlier in females than males. For Texas population, although the median time to anthesis was the same for male and females, the most probable anthesis time (i.e. the bulge) was at an earlier time in females compared to males.

### Plant height and relative growth rate (RGR)

**Fig. 4:**
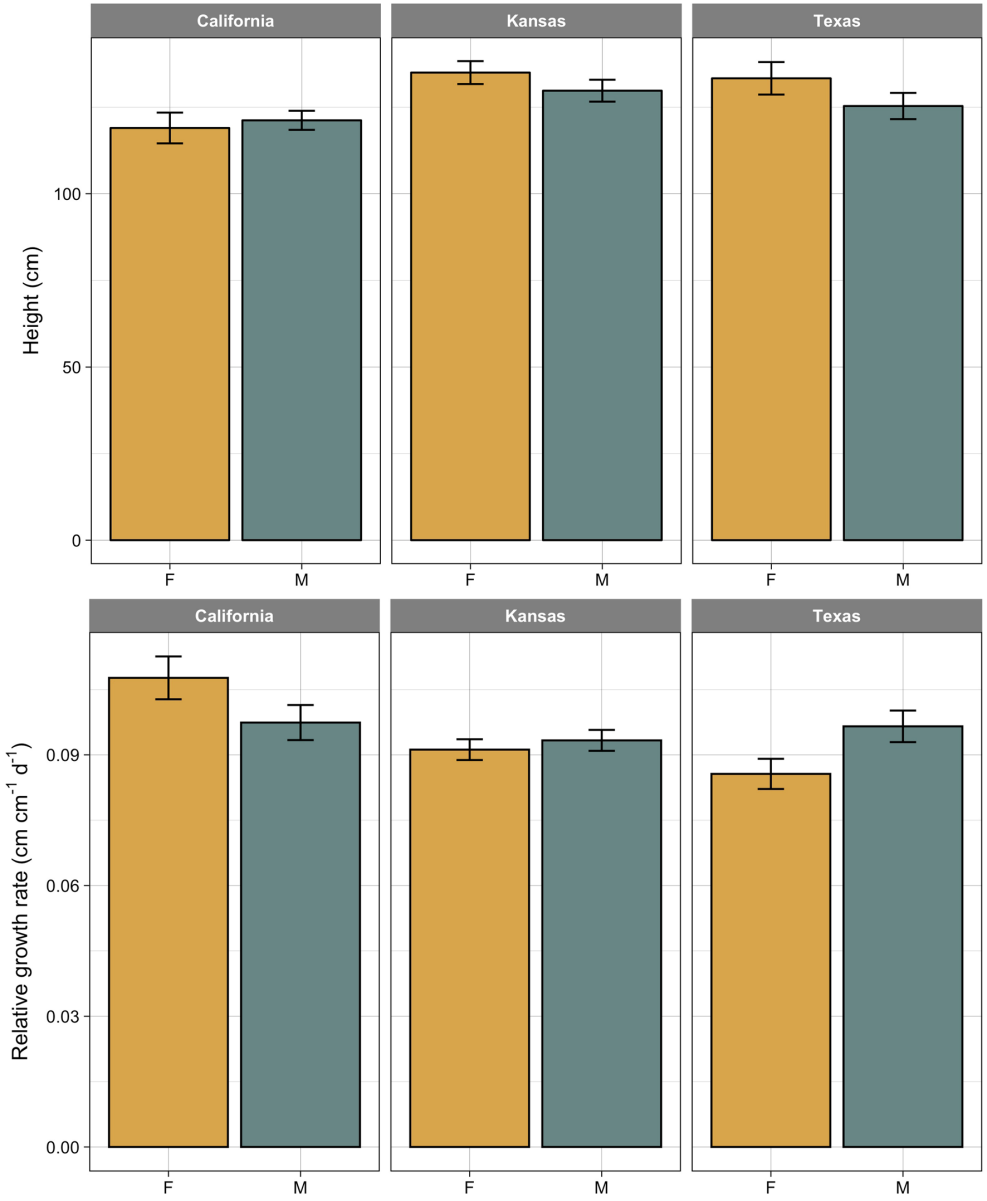
Compression of final plant height (top row) and relative growth rate (bottom row) between female (F) and male (M) plants in three populations of *A. palmeri*. Vertical lines on bars indicate 95% confidence interval

We observed no sex-specific differences in final plant height for the three tested populations (Fig. 4). The only significant sexual dimorphism in growth was related to the height-based RGR in Texas population (*p* value = 0.0332, *df* = 62). At the early stage of growth, males of this population grew tall at a rate that were ~ 13% faster than females. However, the male’s faster initial growth did not translate into significant larger plants as males attained similar stature (125.3 ± 3.78 cm) as with females (133.3 ± 4.69 cm) at the end of growing season.

### Experiment 2. Flowering synchrony in response to water stress

#### Time to flowering and anthesis

**Fig. 5:**
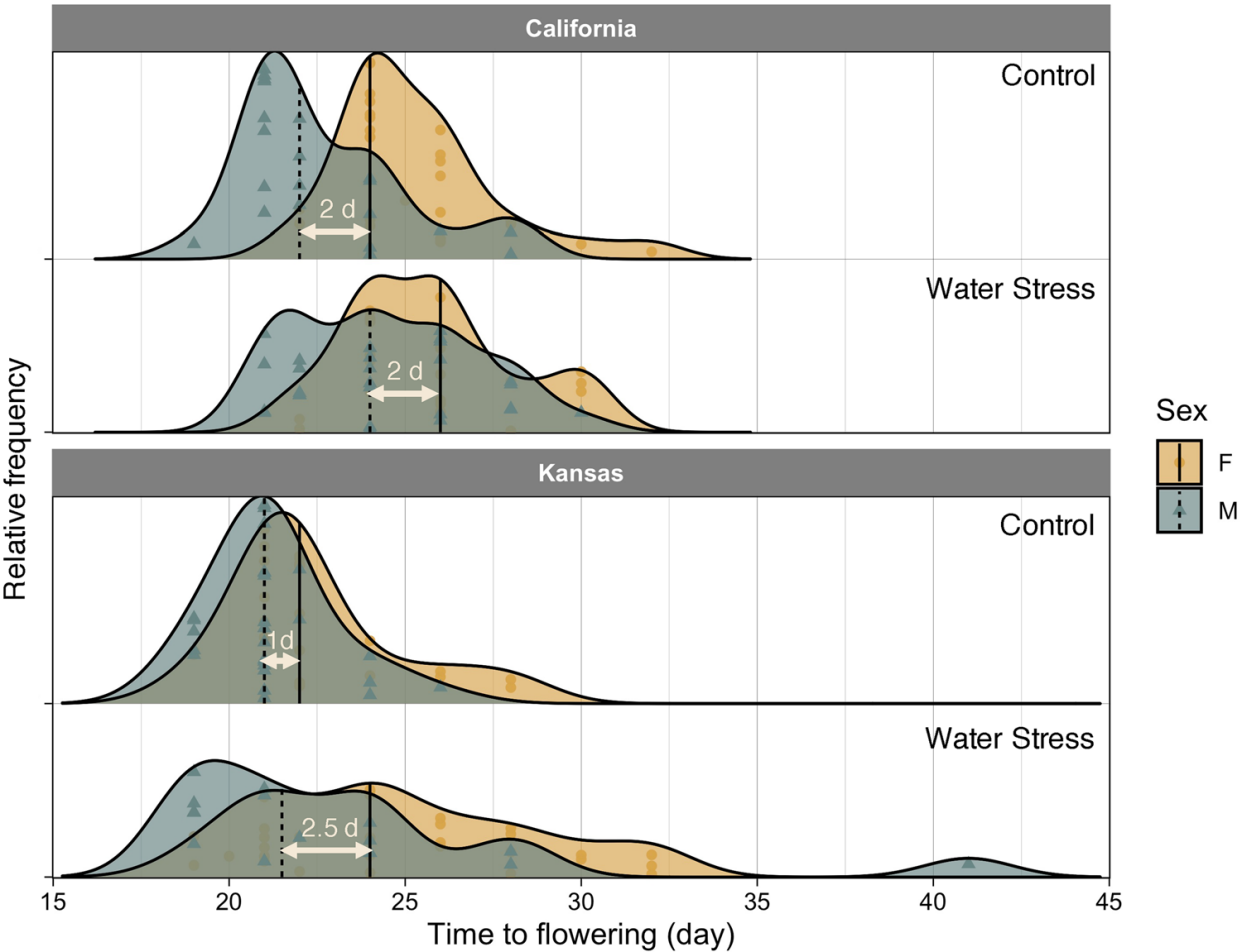
Frequency distribution of time to flowering (emergence of inflorescence) in female (F) and male (M) plants of two *A. palmeri* populations grown under normal watering (control) or water stress conditions. Vertical lines show the location of median time to flowering while symbols indicate observed data. The differences in median flowering time between the two sexes are shown with horizontal arrows

**Fig. 6:**
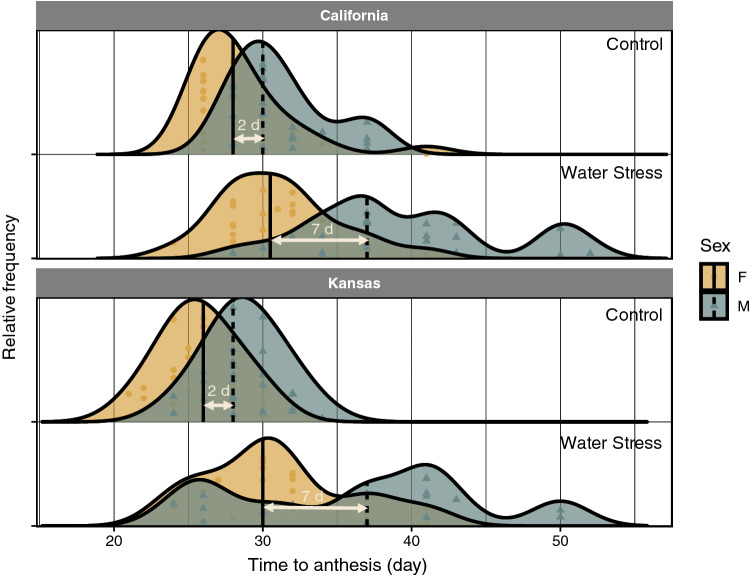
Frequency distribution of time to anthesis in female (F) and male (M) plants of two *A. palmeri* populations grown under normal watering (control) or water stress conditions. Vertical lines show the location of median time to flowering while symbols indicate observed data. The differences in median flowering time between the two sexes are shown with horizontal arrows

Flowering phenology was recorded for plants from California and Kansas populations grown under different irrigation regimes. Similar to *Experiment 1*, the initiation of flowering happened earlier in males than females under both water stress and control conditions (Fig. 5). Water stress slightly delayed flowering, almost to the same extent, in both male and female plants giving rise to a more temporally staggered flowering pattern as compared with the control. Contrary to flowering, anthesis in female plants of *A. palmeri* preceded males (Fig. 6). Sex-differential responses to water stress were observed with the timing of anthesis; water stress distorted the frequency distribution of anthesis timing to a larger extent in males than females (Fig. 6). Time to anthesis nearly followed a unimodal distribution in both sexes when plants received normal irrigation but became multimodal and “wavy” when plants were exposed to water stress. Anthesis was much synchronized between male and female plants of *A. palmeri* under normal watering but the two sexes became more separate (i.e. less overlapping in anthesis) with water stress. In both populations, the difference between males and females in median time to anthesis (arrows in Fig. 6) was only two days in control but significantly increased (*p* value < 0.0001) to seven days in water-stressed plants.

## Discussion

Reproductive synchrony is crucial for the successful mating of dioecious species (Barrett and Hough 2013). Asynchrony in the degree of flowering overlap, i.e. release of male pollens and receptiveness of the female stigmas, will reduce the probability of reproductive success. A synchronous anthesis between male and female plants observed with Texas population resembles the results of Giacomini et al. (2014) who found no differences in days to first flowering (anthesis) between male and female plants of *A. palmeri*. In our study, for females, regardless of the population, the length of time from the appearance of inflorescence to anthesis (visible stigma) was no more than 4–5 days compared with a 11-day time period needed for males to develop mature anthers following the initiation of flowering. The male’s earlier flowering may suggest a protandrous flowering habit in *A. plameri*; however, when we investigated the timing of anthesis, which might be regard as the effective flowering event, we arrived at an opposite conclusion. This observation suggests that *A. palmeri* should be classified as a protogynous species as its pistillate flowers mature earlier than the staminate ones (Fig. 3).

Water stress delayed flowering and anthesis in *A. palmeri* but contrary to our initial hypothesis, the timing of flowering/anthesis in females was less sensitive to stress than males (Figs. 5, 6). The prevalence of protandry in dioecious species has been attributed to the greater cost of reproduction incurred by females (Lloyd and Webb 1977; Forrest 2014). As females need to produce seeds, they may undergo an extended period of resources accumulation to ensure sufficient assimilates will be available for allocation to seeds (Lloyd and Webb 1977; Purrington and Schmitt 1998). Korres et al. (2017) showed that under nutrient deficiency and light stress female plants of *A. palmeri* flowered 6–8 days earlier than male plants, a flowering pattern that is very similar to the results showed in our study from Kansas and California population grown under water stress. However, Korres et al. (2017) do not explicitly define “flowering” stage and we are not sure if our “anthesis” represents the same reproductive stage as with the “flowering” stage in Korres et al. (2017). Plants experiencing stressful environments often accelerate their transition to reproductive stage; perhaps as a potential adaptive strategy to hasten seed production in a timely manner prior to resource disappearance (Wada and Takeno 2010). However, depending on the intensity and duration of stress and plant development stage, stress may produce an opposite response (i.e. delay the flowering) by slowing the plant metabolism (Cho et al. 2017). The delay in flowering of *A. palmeri* observed in Korres et al. (2017) and our study might have resulted from a slower metabolism and lower rate of assimilates accumulation under drought. However, contrary to our initial hypothesis, the timing of flowering/anthesis in females was less sensitive to stress than males. As a result, stress was proposed to exert a stronger effect on the flowering phenology of males than that of females; a hypothesis that was not supported by our data perhaps because of male’s higher propensity for sex conversion. Further, Montgomery et al. (2019) also found that males are heterogametic in *A. palmeri*. These observations suggest that male plants of *A. palmeri* may only have the propensity for sex conversion.

Our study clearly demonstrated that water stress can result in temporal separation of the two sexes in *A. palmeri*, offering a potential means for ecological management of this weed and perhaps other dioecious weeds. Asynchrony in timing of anthesis can reduce the chance of cross-pollination and hence reduce seed production in unisexual weeds, such as *A. palmeri* and common waterhemp (*A. tuberculatus* (Moq.) J. D. Sauer), two dioecious troublesome weeds in the US (van Wychen 2017). Regulated deficit irrigation (RDI) has been proposed to address the water scarcity problem in California (Stewart et al. 2011). In RDI system, the crop is irrigated below the evapotranspiration (ET) requirements so that the crop experiences moderate water stress without any significant reduction in yield. The RDI system not only helps with water saving but may also serve as a weed management practice by decreasing the anthesis overlap *A. palmeri*. Further, summer crops present differences in water use efficiency, thus, providing a tool to limit water availability for *A. palmeri* plants as a summer annual weed. For instance, crops, such as cotton and corn, require higher water availability for plant growth, while in sorghum crops, less frequent irrigation is required for successful crop production (Erbacher et al. 2020). Efforts across the world to generate new transgenic crops with enhanced tolerance to water stress to meet the challenges of future climate change may also be beneficial for the proposed weed management strategy (Mittler and Blumwald 2010).

While the direct (negative) effect of water limitation on seed production is well understood, our study suggests a novel mechanism through which water stress (and perhaps other stressors) can reduce seed production indirectly by exacerbating the flowering/anthesis mismatch between the sexes in a dioecious weed. Future work should attempt to disentangle the direct effects of environmental stressors form their indirect effects on seed production.

### *Author contribution statement*

MBM, MM and SO conceived and designed research. MBM, MM and SO conducted experiments. MBM analyzed the data. MBM and MM wrote the manuscript. All authors read and approved the manuscript.
